# Clusterin: full-length protein and one of its chains show opposing effects on cellular lipid accumulation

**DOI:** 10.1038/srep41235

**Published:** 2017-01-25

**Authors:** Suvarsha Rao Matukumalli, Ramakrishna Tangirala, C. M. Rao

**Affiliations:** 1 CSIR- Centre for Cellular and Molecular Biology, Hyderabad, 500007, India

## Abstract

Proteins, made up of either single or multiple chains, are designed to carry out specific biological functions. We found an interesting example of a two-chain protein where administration of one of its chains leads to a diametrically opposite outcome than that reported for the full-length protein. Clusterin is a highly glycosylated protein consisting of two chains, α- and β-clusterin. We have investigated the conformational features, cellular localization, lipid accumulation, *in vivo* effects and histological changes upon administration of recombinant individual chains of clusterin. We demonstrate that recombinant α- and β-chains exhibit structural and functional differences and differ in their sub-cellular localization. Full-length clusterin is known to lower lipid levels. In contrast, we find that β-chain-treated cells accumulate 2-fold more lipid than controls. Interestingly, α-chain-treated cells do not show such increase. Rabbits injected with β-chain, but not α-chain, show ~40% increase in weight, with adipocyte hypertrophy, liver and kidney steatosis. Many, sometimes contrasting, roles are ascribed to clusterin in obesity, metabolic syndrome and related conditions. Our findings of differential localization and activities of individual chains of clusterin should help in understanding better the roles of clusterin in metabolism.

Clusterin is a predominantly secreted glycoprotein consisting of two chains–α-clusterin (α-Clu) and β-clusterin (β-Clu) that are linked by 5 disulphide bonds. It is expressed in several tissues and is present in the extracellular space and various body fluids^1^. Different proteoforms of clusterin are known to exist, and mutations in the protein might lead to its altered localization and functions in the cell^2^.

Since its discovery as a cell-aggregating factor found in ram testis fluid^3^, several roles have been ascribed to clusterin such as complement inhibition^4^, regulation of inflammation^5^, lipid transport^6^, apoptosis^7^, cell differentiation^8^, appetite regulation^9^ and protein quality control in the extracellular space^10^. Clusterin has been shown to exhibit chaperone-like activity and prevents the chemically-induced and heat-induced amorphous aggregation^11^,^12^ as well as amyloid aggregation^13^,^14^ of proteins *in vitro*. Although its exact role in many conditions is not very clear, it is implicated in neurodegenerative disorders such as Alzheimer’s^15^, several cancers^16^, autoimmune disorders and chronic inflammatory disorders^5^. Clusterin has been identified as a biomarker of Alzheimer’s disease in several genome-wide association studies^17^,^18^, it is associated with Aβ-plaques in Alzheimer’s and it has been found to inhibit the amyloid fibril formation of Aβ *in vitro*^15^.

Clusterin was shown to modulate the activity of leptin and act as an anorexigenic molecule in animals^9^. Lipoprotein-associated clusterin binds to ghrelin, a peripheral orexigenic peptide^19^. Clusterin has been shown to bind to promoter regions of Sterol Regulatory Element Binding Protein-1C (SREBP-1C), a master regulator of several lipid metabolic pathways, regulating its expression^20^ and inhibiting hepatic lipid accumulation^21^. These studies indicate a protective role for clusterin in metabolic disorders. Indeed, commonly known polymorphisms in clusterin gene are positively correlated with the risk of type-II diabetes (T2DM)^22^. However, there are unclear reports on the correlation between serum clusterin concentration and T2DM^23^,^24^,^25^. Similarly, unclear reports also exist for correlation between levels of clusterin and obesity^25^,^26^. Certain studies have indicated that a specific proteoform of clusterin might be better correlated with T2DM than other isoforms^27^,^28^.

Different proteoforms of clusterin including nuclear clusterin, cytosolic clusterin, and variably glycosylated forms of clusterin have been found to exist naturally. Several of these proteoforms have been shown to have different functions. Evidence suggests that even the two chains of clusterin might exist separately. β-Clu was shown to be associated with the acrosomal membrane of healthy sperms^29^, whereas α-Clu has been shown to have differential immunolocalization in the light-damaged retinas of rats^30^. These studies indicate a functional role for the individual chains of clusterin. Despite evidence for the independent existence of the two chains, their structure and function has not been investigated as yet.

In the current study, we demonstrate that the individual, recombinant α- and β-chains exhibit structural and functional differences and differ in their sub cellular localization. Interestingly, while full-length clusterin is known to decrease cellular lipid accumulation, we find that β-Clu, in contrast, causes a significant increase in cellular lipid accumulation.

## Results

### Structural Characterization

**Figure 1:**
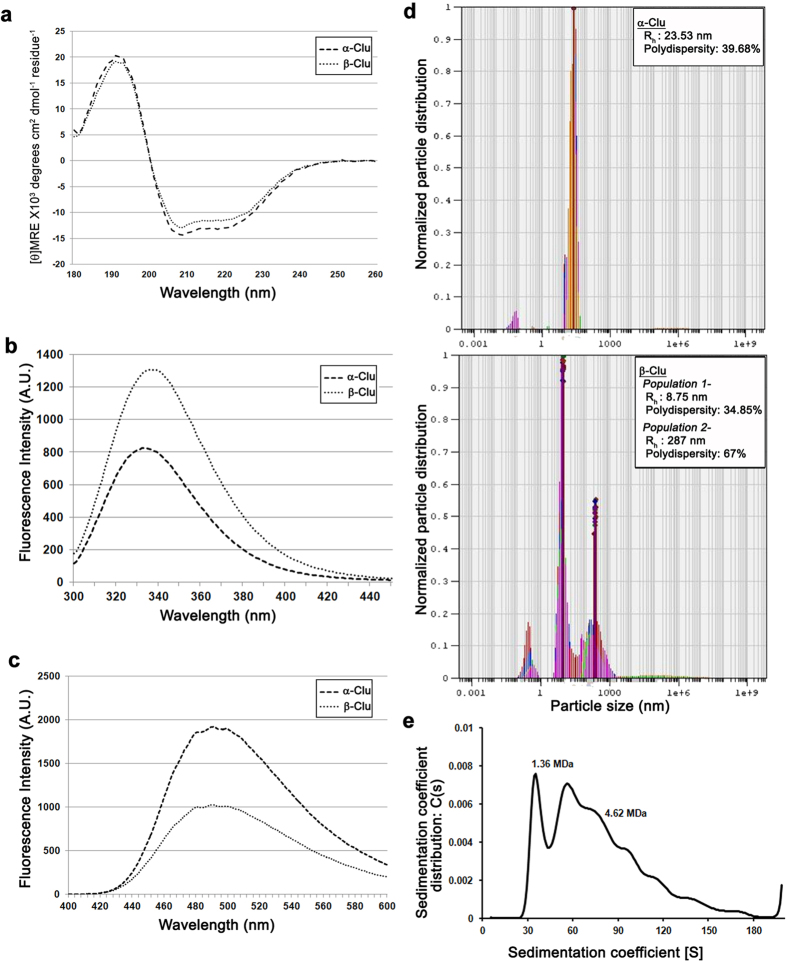
Biophysical characterization of α-Clu and β-Clu. (**a**) Far-UV CD spectra of α-Clu (- - - -) and β-Clu (….). [θ]MRE represents the mean residue ellipticity. (**b**) Intrinsic tryptophan fluorescence spectra of 0.2 mg/ml of α-Clu (- - - -) and β-Clu (….) in PBS. Excitation wavelength was set at 295 nm and the excitation and emission band passes were set at 2.5 nm. (**c**) Fluorescence spectra of the hydrophobic fluorescent probe, bis-ANS, alone (-----) or in the presence of α-Clu (- - - -) or β-Clu (….). The excitation wavelength was set at 390 nm and excitation and emission band passes were set at 2.5 nm. (**d**) DLS population distribution for α-Clu (i) and β-Clu (ii). The distribution obtained is an average of 3 sets of 20 acquisitions each. The abscissa indicates the hydrodynamic radius of the molecule, ordinate is relative abundance of the molecules and the area under the curve indicates the polydispersity. (**e**) Distribution of sedimentation coefficients of α-Clu. The molecular masses of the species determined from the sedimentation velocity data by solving Lamm’s equation are also indicated.

We have expressed and purified the α- and β-chains of clusterin to homogeneity with a yield of ~25 mg purified protein per litre of bacterial culture. In conformity with the nomenclature used earlier^31^, we refer to the two chains as α-Clu and β-Clu. The far-UV CD spectra of α-Clu and β-Clu are very similar and exhibit minima at 218 nm and 208 nm (Fig. 1a). We analyzed the far-UV CD spectra using the CDNN program^32^. Our results indicate that α-Clu has 39.1% α-helix, 13.1% β-sheet, 16.4% β-turns and 24.6% random coil, whereas β-Clu has 36% α-helix, 15.3% β-sheet, 16.8% β-turns and 27.2% random coil. Thus, the CD study indicates significant α-helical structure for both α-Clu and β-Clu. A BLAST analysis of the two chains shows that they have 23% identity and 51% similarity with each other.

The fluorescence emission spectra of α-Clu and β-Clu upon excitation at 295 nm exhibit emission maximum at 333 nm and 338 nm respectively (Fig. 1b), indicating that tryptophan residues in both proteins are in a hydrophobic environment, the tryptophans in α-Clu being in a slightly more hydrophobic environment than those in β-Clu.

We have used bis-ANS to probe the hydrophobic surfaces of α-Clu and β-Clu. The fluorescence intensity of bis-ANS is known to increase several-fold, and its emission maximum exhibits a blue shift, when bound to hydrophobic surfaces of a protein^33^. We observed an increase in the fluorescence intensity of bis-ANS and a blue shift in its emission maximum to 498 nm when bound to α-Clu or β-Clu, suggesting that they have exposed hydrophobic surfaces. However, the intensity of β-Clu-bound bis-ANS fluorescence is almost half of that of α-Clu-bound bis-ANS, indicating that surface hydrophobicity of β-Clu is lesser than that of α-Clu (Fig. 1c).

DLS studies showed that α-Clu forms a single polydisperse population of oligomers with an R_h_ of ~23.5 nm. β-Clu formed 2 populations with one population having an R_h_ of 8.75 nm and the other with an average R_h_ of 287 nm (Fig. 1d).

Sedimentation velocity measurement study (Fig. 1e) shows that α-Clu exhibits a distinct peak with a sedimentation coefficient of 36.8 S and an approximate molecular mass of about 1.4 MDa. Considering the sequence-based theoretical molecular mass of the subunit as 24 kDa, the 1.4 MDa population of α-Clu forms oligomeric assemblies with about 60 subunits per oligomer. In addition, the distribution of sedimentation coefficients shows the presence of other populations of α-Clu with sedimentation coefficients of 83S and higher. This result suggests that α-Clu forms large oligomers as is also evident by the DLS results. β-Clu, on the other hand, formed very large particles and could not be analyzed by sedimentation velocity experiments.

### Chaperone activity

**Figure 2:**
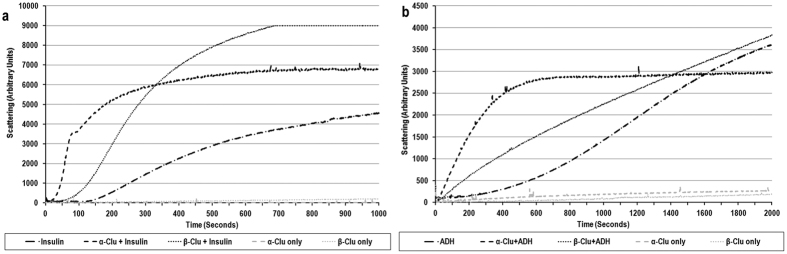
Chaperone activity of α-Clu (- - -) and β-Clu (…) against (**a**) DTT induced aggregation of insulin (-.-.-) and (**b**) Heat induced aggregation of yeast ADH (-.-.-). Control traces with α-Clu alone (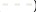) and β-Clu alone (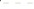) are shown in both panels.

We used heat-induced aggregation of yeast ADH as well as the DTT-induced aggregation of insulin as model systems to assay chaperone-like activity of α-Clu and β-Clu. Both α-Clu and β-Clu failed to prevent the DTT-induced aggregation of insulin at a 1:1 (w/w) ratio (Fig. 2a). Under the assay conditions, α-Clu or β-Clu alone did not undergo any aggregation. In fact, the aggregation of insulin in the presence of the proteins was higher than that of insulin alone, suggesting that α-Clu and β-Clu co-precipitated out with insulin. Yeast ADH aggregates at a temperature of 48 °C. Figure 2b shows the effect of α-Clu and β-Clu on the heat-induced aggregation of yeast ADH. At a target protein to chaperone ratio of 1:1 (w/w), both proteins do not offer any protection against aggregation. Our results show that unlike full-length serum clusterin, the individual chains of clusterin do not have any chaperone activity *in vitro* against the model systems tested.

### Sub cellular localization

**Figure 3:**
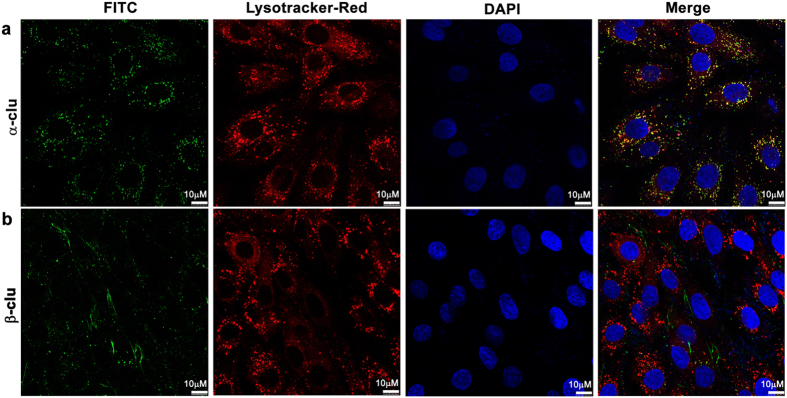
Sub cellular localization of α-Clu and β-Clu. C2C12 cells were incubated with FITC- α-Clu or FITC-β-Clu for 24 hours, followed by incubation with Lysotracker Red for 1 hour. Cells were visualized on Leica SP8 Confocal platform. Excitation of He-Ne diode of 405 nm for DAPI and Argon laser lines of 488 nm and 560 nm for FITC and Lysotracker respectively were used. (**a**) α-Clu; (**b**) β-Clu. Represented images are single mid-sections of observed fields. Scale bar represents 10 μm.

We incubated C2C12 cells with FITC-labelled α-Clu or β-Clu, and observed their sub cellular localization using confocal microscopy. FITC-labelled α-Clu showed a speckled distribution in the cytoplasm of the cells. FITC-labelled α-Clu did not localize to the mitochondria, the endoplasmic reticulum (data not shown) or the nucleus (Panels 3 and 4 in Fig. 3a). Upon co-staining with Lysotracker red, FITC- α-Clu co localized with the lysosomes with a degree of co localization of 94.17% and a Pearson’s correlation coefficient of 0.925 (Fig. 3a).

Interestingly, FITC-labelled β-Clu did not localize to the lysosomes. FITC-labelled β-Clu showed 2 distinct distributions in the cell - a speckled distribution throughout the cell which did not co localize with the ER, nucleus, mitochondria, or the lysosomes, and a thread-like pattern at the cell peripheries and on cytoplasmic projections (Fig. 3b).

### Accumulation of neutral lipids in β-Clu-treated cells

**Figure 4:**
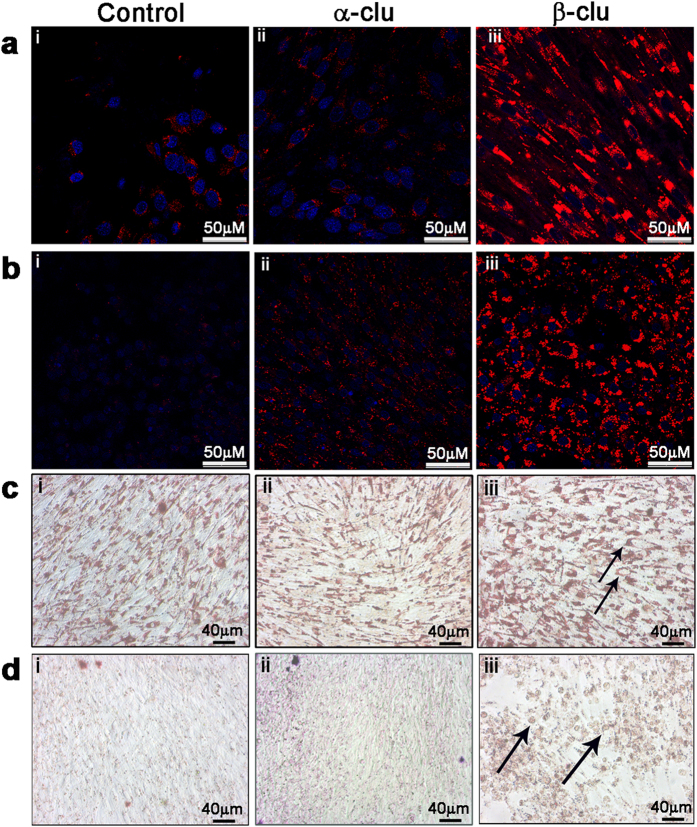
Lipid accumulation in cell lines. Control cells (i), α-Clu-treated cells (ii) and β-Clu-treated cells (iii) stained with Nile Red (Panel **a**: C2C12; Panel **b**: F111) and Oil Red O (Panel **c**: C2C12; Panel **d**: F111). Solid black arrows in **c** and **d** represent morphological changes observed in the cells upon incubation with β-Clu. Scale bar in panels **a** and **b** represent 50 μm. Scale bars in panels **c** and **d** represent 40 μm.

We have studied the effect of extracellular treatment of α-Clu and β-Clu on the intracellular accumulation of lipids in C2C12 cells. Nile red, a selective probe for accumulation of intracellular lipid droplets, becomes strongly fluorescent upon binding to lipids^34^. We stained cells with Nile red and visualized them by confocal microscopy. Both control C2C12 cells and α-Clu-treated cells show feeble Nile red fluorescence under the experimental conditions (Fig. 4ai,aii). Cells treated with β-Clu show a striking increase in the Nile red fluorescence (Fig. 4aiii), indicating intracellular accumulation of neutral lipids. Similar results were obtained with the rat fibroblast cell line, F111 (Fig. 4b).

**Figure 5:**
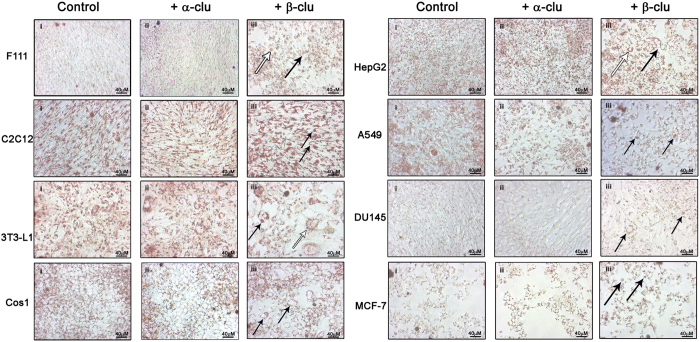
Lipid accumulation in cells upon β-Clu administration is a general phenomenon. Oil red O staining of control cells (i) and cells treated with α-Clu (ii) and β-Clu (iii). Morphological changes are represented by arrows. Hypertrophy in 3T3-L1 preadipocyte cells and HepG2 and F111 cells are represented by white arrows. Changes in cellular shape in the cells treated with β-Clu are denoted by black arrows. Scale bar represents 40 μm.

Another widely used reporter for intracellular lipid accumulation, Oil Red O (ORO)^35^, also showed significant accumulation of intracellular lipid droplets in β-Clu-treated C2C12 and F111 cells compared with control cells. However, cells treated with α-Clu did not show accumulation of lipids (Fig. 4c,d). We have also used cancer cell lines such as MCF-7, DU145 and A549, and other cell lines such as HepG2, Cos1 and 3T3-L1 and obtained similar results (Fig. 5). Apart from lipid accumulation, we also observed morphological changes such as rounding and hypertrophy of certain cells upon treatment with β-Clu (Fig. 5). Our results show that β-Clu-associated intracellular lipid accumulation is a general phenomenon.

**Figure 6:**
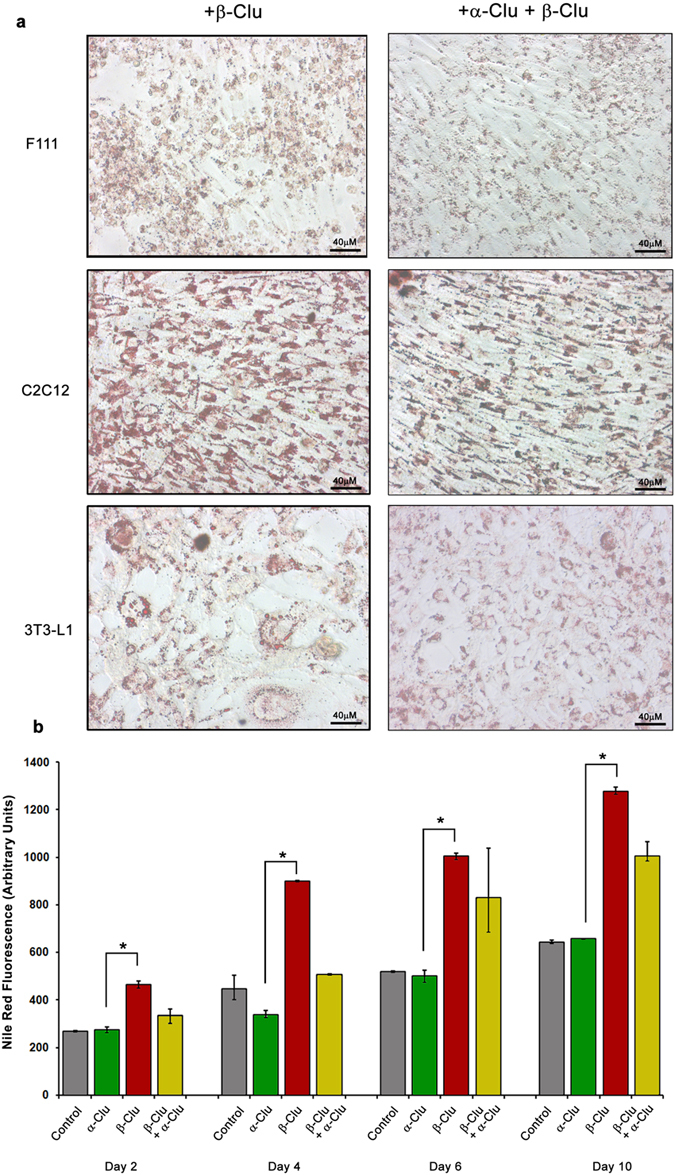
Morphological changes and time-dependant accumulation of lipids in cells treated with clusterin chains. (**a)** Morphology of cells treated with (i) β-Clu alone and (ii) both α-Clu and β-Clu at equimolar ratios. (**b)** Time-dependant increase in lipid accumulation in β-Clu-treated cells over control and α-Clu treated cells and rescue of phenomena upon addition of equimolar ratios of α-Clu and β-Clu, quantified by FACS (*p = 0.008, two-tailed T-test).

We have incubated C2C12, F111 and 3T3-L1 cells with equimolar concentrations of α-Clu and β-Clu (10 μM each) and studied the accumulation of lipids in them using ORO. We observed a marked reduction in the lipid accumulation in cells when compared with cells treated with just β-Clu (Fig. 6). Morphological changes observed with β-Clu administration were also markedly reduced (Fig. 6a).

In order to study the time-dependence of lipid accumulation, we have incubated C2C12 cells with α-Clu, β-Clu or an equimolar ratio of both proteins (10 μM each) for 2, 4, 6 or 10 days and quantified lipid accumulation (using Nile red) as mean fluorescence intensity per cell. Accumulation of lipid increased marginally in the α-Clu-treated cells, but they showed mean fluorescence intensity similar to controls at every time point. However, β-Clu-treated cells showed a consistent 2-fold increase in accumulation of lipid at every time point over that in control and α-Clu-treated cells (Fig. 6b). Interestingly, co-administration of α-Clu and β-Clu causes a marked reduction in intracellular lipid accumulation. The time course analysis illustrates that this change was evident as early as Day 2 of protein administration, indicating a strong dominant effect of α-Clu over β-Clu. However, at later time points, the effect of α-Clu was not as pronounced and it did not completely abrogate the lipid accumulation phenomenon caused by β-Clu (Fig. 6b).

### Weight increase in animals injected with β-Clu

**Figure 7:**
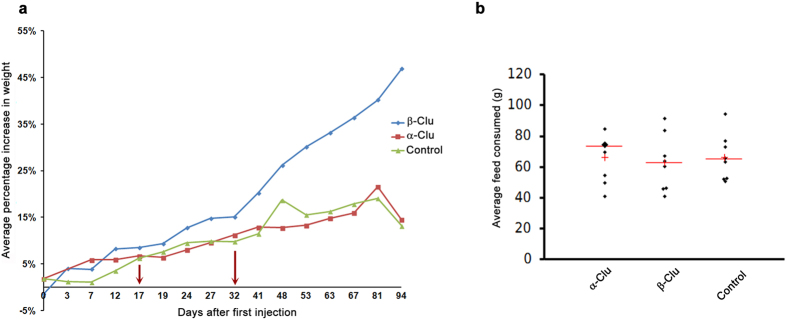
Weight change and feed intake of rabbits injected with α-Clu or β-Clu. (**a**) Average percentage increase in weight of rabbits injected with β-Clu over controls and α-Clu-injected rabbits (n = 8). Red arrows indicate times of booster injections. (**b**) Average daily feed intake in controls (n = 8) and rabbits injected with α-Clu (n = 8) or β-Clu (n = 8). Red horizontal bar represents mean value. Red + represents median.

We studied the effect of injecting α-Clu or β-Clu subcutaneously in New Zealand White Rabbits. Weight of β-Clu-injected rabbits, but not α-Clu-injected rabbits, increased ~40% from 15 days post injection to up to 90 days after injection (Fig. 7a) over that of controls (n = 8). Interestingly, there was no considerable change observed in the average feed consumed in all the 3 groups of animals (Fig. 7b).

### Histological examination of animal tissues

**Figure 8:**
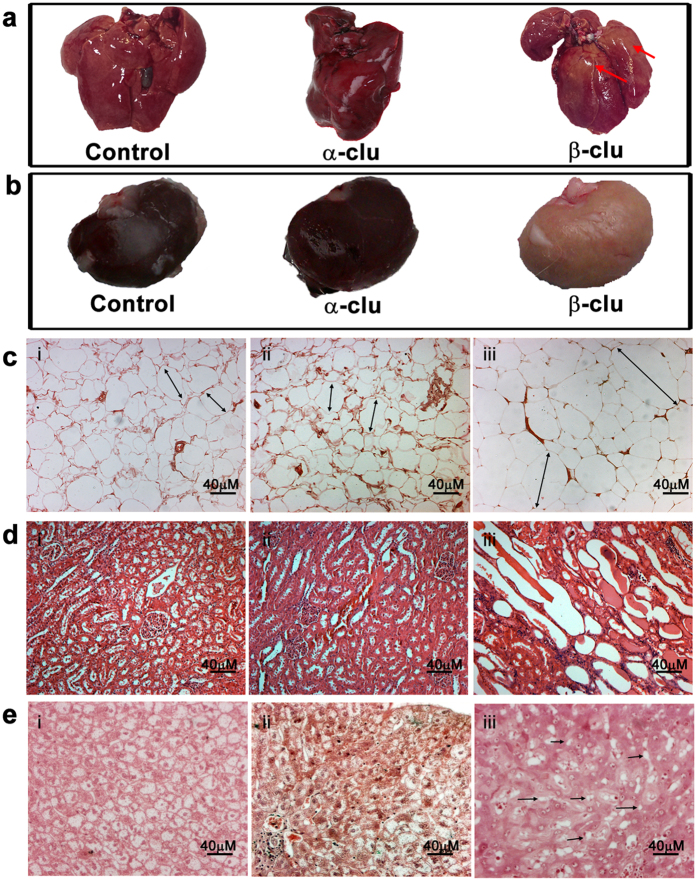
Changes in organs and tissues of rabbits injected with β-Clu. (**a**) Fatty changes in liver of β-Clu-injected rabbit evident as focal patchy yellowish discoloration, depicted by red arrows. (**b**) Fatty changes in kidney of β-Clu-injected rabbit depicted by pale colour of the kidney compared to control. Histological examination of (i) control rabbits, (ii) α-Clu-injected rabbits and (iii) β-Clu-injected rabbits; (**c**) Adipose tissue, (**d**) Kidney, (**e**) Liver. Double sided arrows indicate relative hypertrophy in β-Clu-injected rabbits with respect to control and α-Clu-injected rabbits. Black arrows indicate extensive steatosis in liver tissues of β-Clu injected rabbits. Scale bar represents 40 μm.

Animals were sacrificed and their organs were isolated and fixed in 10% Neutral Buffered Formalin (NBF). We observed discoloration in the liver (Fig. 8a) and kidney (Fig. 8b) of β-Clu-injected animals indicative of fatty changes. Histological examination of paraffin-fixed tissues showed mild hypertrophy in the subcutaneous adipose tissue of β-Clu-injected animals. Control animals and α-Clu-injected animals did not show such changes in the subcutaneous adipose tissue (Fig. 8c). We also observed extensive steatosis, visible as empty spaces in the kidney tissue (Fig. 8diii), and a distinct lack of cell wall definition with a marked foamy appearance in liver cells (Fig. 8eiii) of β-Clu-injected animals^36^. The fatty changes and steatosis were absent in animals treated with α-Clu (8d ii, 8e ii) and controls (8d i, 8e i).

## Discussion

Clusterin exists in many forms in the cell, and its biological functions depend upon its localization, glycosylation and the splice variant expressed. Individual chains are also reported to exist independently^29^,^30^. Thus, understanding the structural and functional differences of the two chains has physiological significance. We have expressed and purified individually, the α-Clu and β-Clu chains of human clusterin, and studied their structure and role with respect to lipid accumulation *ex vivo* and *in vivo*. Both α-Clu and β-Clu show appreciable α-helical content (39 and 36% respectively). However, compared to the α-helical content of full-length, secretory clusterin (~62% α-helix, 7% β-sheet, ~12% turns and 20% random coil), which consists of both the chains linked by 5 disulphides, their α-helical content is significantly less^37^. This result suggests that long range interactions stabilize the α-helicity of some of the regions in the full length clusterin. Though the two chains of clusterin exhibit similar secondary structural content, they vary in their surface hydrophobicity, tryptophan solvent accessibility and oligomeric size.

Serum clusterin has been shown to exist in solution as a polydisperse mixture of oligomers of different mass ranges. The reported hydrodynamic diameter of the whole clusterin molecule as recorded by DLS is 15 to 20 nm^38^. Also, gel filtration chromatography and sedimentation velocity experiments suggest that full-length clusterin at physiological pH exists as a polydisperse population consisting of monomers, dimers, tetramers and some very high molecular weight oligomers in solution^11^,^39^. Studies by Stewart *et al*. report that de-glycosylated clusterin shows a propensity to form higher oligomers than wild-type glycosylated secreted clusterin^40^. In our studies, we noticed that both α-Clu and β-Clu form higher order oligomers with large hydrodynamic radii and large molecular weights as observed in sedimentation velocity experiments. Two oligomeric populations were found to exist for β-Clu in DLS, one consisting of rather small oligomers and the other consisting of very large oligomers. Therefore, the natural propensity of the individual chains to assemble into higher-order oligomers is similar to that observed for the full-length protein in physiological conditions. However, lack of glycosylation might be responsible for the formation of very large oligomers for both the chains, particularly in the case of β-Clu, which could not be analyzed by sedimentation velocity experiments.

Clusterin has been shown to have chaperone-like activity against heat-induced aggregation and DTT-induced aggregation of model proteins^11^. We assayed the chaperone-like activity of α-Clu and β-Clu in these two systems of aggregation. Interestingly, we observe that neither of the chains exhibits any significant chaperone-like activity.

It has been suggested that full-length clusterin binds to client proteins through the hydrophobic patches present on the protein and chaperones them from aggregation^41^. In the case of α-Clu and β-Clu, we see a similar result in that both proteins have high degree of surface hydrophobicity and form very large oligomers when compared to full-length clusterin but lack the chaperone activity shown by the intact, full-length clusterin.

Though Stewart *et al*. have reported that deglycosylation of clusterin does not significantly affect the chaperone activity of the protein^40^, another study by Rohne *et al*. showed that complete deglycosylation of clusterin, in the presence of DTT, causes up to 90% loss in the chaperone activity of the protein^37^. Intriguingly, clusterin is reported to have two levels of glycosylation; core glycosylation involving mannose moieties and further glycosylation of these residues with higher oligosaccharides^42^,^43^. It was shown by Rohne *et al*. that hypoglycosylated clusterin, having just the core glycosylation, had significant chaperone-like activity and even completely deglycosylated clusterin showed minimal chaperone activity^37^. However, in our experiments we observe that both α-Clu and β-Clu co-aggregate with insulin when subjected to DTT-induced aggregation. The aggregation profile of yeast ADH also changes significantly when incubated along with the proteins, with ADH undergoing very rapid aggregation.

Unpublished data from our laboratory also showed that the individual chains α-Clu and β-Clu did not exhibit any protection against the amyloid aggregation of β2-microglobulin and α-synuclein (Sultan *et al*., unpublished). Full-length clusterin, on the other hand, is shown to protect against the amyloid aggregation of several model proteins^13^. Taken together, the results from the present study and the previous unpublished results from our laboratory indicate that the two chains of clusterin do not protect against either the amorphous or amyloid aggregation of proteins, unlike full-length clusterin. Above studies suggest that regions from both the chains might be required for the chaperone action of the full-length clusterin. As mentioned above, completely deglycosylated clusterin has been shown to have minimal chaperone activity. Therefore, another possible explanation for the lack of chaperone function of the two chains might be the absence of even the core glycosylation in our protein preparations. The role of glycosylation in the chaperone-like activity of clusterin can be investigated by co-refolding or chemically crosslinking the recombinant non-glycosylated chains to obtain the full-length clusterin and probing for its chaperone-like activity.

While many effects of clusterin are indeed extracellular, it is important to note that in several cases clusterin has intracellular effects such as in the modulation of apoptosis where secretory clusterin and nuclear clusterin bind to different targets causing anti- or pro-apoptotic effects respectively^7^,^44^. Also, Nizard *et al*. have shown that when cells are stressed by treatment with thapsigargin or high extracellular concentrations of KCl, clusterin is retained in the cytosol instead of being secreted^45^. In our study, both chains differ strikingly in their subcellular localization when incubated with cells: α-Clu localized to the lysosomes, whereas β-Clu showed speckled localization in the cytoplasm and fibre-like morphology along the cell periphery. A trivial explanation for the observed peripheral localization of β-Clu on cells could be that the larger sized population of β-Clu could not enter the cells. However, this may be unlikely as the cells were extensively washed before fixation. Moreover, previous studies have shown that β-Clu localizes to the sperm acrosomal membrane^29^, indicating that clusterin has a propensity to associate with the cellular membrane. Membrane association for clusterin has also been reported in earlier studies^29^,^46^,^47^,^48^. However, which form/part of clusterin mediates its membrane interaction is not known. Based on our present results of peripheral localization of β-Clu as well as earlier studies on sperm acrosomal membrane localization^29^, we speculate that the observed membrane association could be that of β-Clu chain present independently or that of full-length clusterin, mediated through β-Clu. An earlier study showed that expression of GFP-tagged β-Clu in Cos-7 cells resulted in a diffused localization throughout the cell, while that of GFP-tagged α-Clu resulted in its localization in juxtanuclear aggregates (aggresomes)^31^. Our results are markedly different from those reported earlier^31^, and indicate that the observed sub cellular localization of clusterin chains could differ depending on whether they are presented extracellularly (our present study) or expressed intracellularly^31^. Alternately, proteins used in our study are produced recombinantly and hence lack glycosylation while the proteins used in the study by Debure *et al*.^31^ have extensive glycosylation. This might lead to alterations in their structures and hence, alterations in their intracellular functions.

In recent years, various studies have shown a role for clusterin in metabolism. Clusterin can modulate metabolic homeostasis in three different ways: firstly, by regulation of lipogenic pathways directly and indirectly; secondly, by regulating appetite, thereby controlling energy supply and thirdly, by control of several other parameters such as oxidative stress, inflammation and lipid transport in several metabolic disorders.

Clusterin negatively regulates the expression of SREBP-1C, causing a decrease in hepatic lipid accumulation in cell culture as well as in animal models^21^. Also, insulin-stimulated SREBP-1c regulates clusterin expression during lipogenesis^20^. Since full-length clusterin causes a reduction in cellular lipid, we thought that investigating the behaviour of the individual chains might provide important insights for the development of therapeutic strategies against lipid storage disorders.

Our studies show that β-Clu causes an increase in lipid accumulation compared to that in control cells in all the cell lines studied, indicating that β-Clu affects a general phenomenon. However, this effect is not seen in the case of α-Clu-treated or control cells. Interestingly, upon co-administration of both the proteins, we observe a marked reduction in the effect caused by β-Clu. Our results also show that this effect of α-Clu is more pronounced at early time points (2 days and 4 days) rather than at later time points. It should be noted, however, that intact, full-length clusterin was shown, in contrast, to decrease the cellular lipid accumulation.

Morphological changes such as rounding of cell and hypertrophy are seen in the cells treated with β-Clu, which are not seen with α-Clu administration. Also, when α-Clu and β-Clu are administered together, these morphological changes are not evident and cells appear similar to control cells. Hypertrophy and cell shape variations are commonly seen in adipogenic cell lines such as 3T3-L1 that are differentiating into adipocytes^49^. Hence, one of the reasons for the observed morphological changes could be the cellular response of the cells to intracellular lipid accumulation as in the case of adipogenic differentiation. Alternately, β-Clu might be associating with cellular cytoskeletal elements and causing a subsequent change in the cell morphology. A study by Moretti *et al*.^50^ showed that nuclear and secreted forms of clusterin interacted differentially with α-actinin; they found that nuclear, but not secretory, clusterin dismantled the cellular cytoskeleton, changing the cell morphology.

Leptin is known to be an anorexigenic molecule^51^,^52^. An earlier study has shown that clusterin modulates the activity of leptin by binding to it^9^. Clusterin has been shown to facilitate the internalization of leptin into hypothalamic neurons using the LDL receptor-related protein 2^53^, eventually resulting in increased STAT3 activation^54^. The above studies indicate that clusterin would cause a reduction in appetite. Indeed, studies by Kim *et al*.^55^ have shown that administration of full-length clusterin results in reduced feed consumption and a decrease in weight. On the contrary, studies by Zeng *et al*.^56^ have shown that clusterin does not cause a reduction in appetite or weight. Furthermore, studies by Arnold *et al*.^57^ also showed that plasma clusterin levels are not correlated with leptin or weight loss and that clusterin may not be an important modulator of leptin activity. In our study, β-Clu-injected rabbits show an increase in weight but no increase in feed consumption over controls (Fig. 7). Similarly, administration of α-Clu also did not cause any change in feed consumption over controls. We propose that the appetite-modulatory effects of clusterin, if any, might be due to specific proteoform and might also depend on the appropriate conformation of the protein. Our results indicate that β-Clu might modulate pathways involved in energy expenditure rather than energy uptake, leading to alterations in the metabolic homeostasis.

Adipose hypertrophy is known to cause an increase in the concentrations of pro-inflammatory adipokines which eventually leads to inflammation and hepatic steatosis^58^. While α-Clu does not exhibit any deleterious effect, we find that β-Clu causes steatosis. One of the reasons for the development of several metabolic disorders over time is lipotoxicity - the accumulation of lipid and subsequent degeneration of tissues^59^. Histological examination of the tissues of β-Clu-injected rabbits shows a remarkable degree of fatty degeneration or steatosis in the metabolically important organs such as liver and kidney. Such changes were not observed in α-Clu-treated animals or the controls.

Thus, our study shows that β-Clu, but not α-Clu, affects cellular lipid accumulation as well as causes lipotoxicity; neither of the chains has an effect on appetite modulation. Our preliminary *ex vivo* studies on the accumulation of lipids indeed show that the observed deleterious effects of β-Clu can be rescued to a certain degree by the addition of α-Clu along with it. We propose that the individual chains of clusterin function in different ways and the expression of different proteoforms with either α-Clu or β-Clu as a dominant molecule might explain the contradictions reported in the role of clusterin in metabolic regulation. Understanding the differential role of the individual chains might help in elucidating the conflicting reports and also in designing strategies to address metabolic disorders.

## Materials and Methods

### Expression and purification

The α- and β-chains of human clusterin were cloned by amplifying the clusterin cDNA from total cDNA of IMR32 human neuroblastoma cells. The forward (FP) and reverse primers (RP) used for cloning the α-chain of clusterin are: FP-GGG AAT TCC ATA TGG ACC AGA CGG TCT CAG AC and RP-CCG CTC GAG TCA GCG GAC GAT GCG G, and for the β-chain are: FP-GGG AAT TCC ATA TGA GCT TGA TGC CCT TCT CTC C and RP-CCG CTC GAG TCA CTC CTC CCG GTG C (Sultan *et al*., unpublished). *E. coli* strain BL21 (DE3) (Novagen, USA) was transformed with pET- 21a(+) expression vectors containing coding sequences of human α-Clu or β-Clu. The transformed cells were cultured in Luria–Bertani medium at 37 °C in a rotary shaker at 250 rpm. Protein expression was induced with 1 mM Isopropyl β thiogalactoside. Both α-Clu and β-Clu were expressed in the insoluble fraction, with maximum expression observed at 5th hour after induction. Cells were lysed using lysozyme and sonication in the presence of dithiothreitol (DTT). The inclusion bodies obtained were washed with 0.1% tritonX-100, dissolved in 20 mM Tris buffer (pH 9 for α-Clu, pH 7.2 for β-Clu) containing 8 M urea and 2 mM DTT and loaded on a DEAE-Sepharose ion exchange column. Upon elution with 20 mM NaCl, fractions containing the protein were pooled and dialysed against Tris buffer (pH 7.4) containing 100 mM NaCl and 1 mM EDTA in order to facilitate refolding of the protein. The proteins were then concentrated, buffer exchanged to phosphate buffered saline (PBS) at pH 7.4 and stored at 4 °C for further experiments. The purity of the obtained proteins was investigated using SDS-PAGE.

### CD spectroscopy

CD spectra of α-Clu and β-Clu in PBS (pH 7.4) were recorded on a Chirascan plus CD spectropolarimeter (Applied Photophysics, UK). Far-UV CD spectra were recorded with a 0.2 mg/ml sample of protein in a 0.1 cm path length cuvette. Temperature was maintained at 25 °C during all measurements. Buffer spectra recorded under the same conditions were subtracted from protein spectra to obtain the final measurements. The spectra reported are the average of 4 scans. The observed ellipticity values were converted to mean residue ellipticities (MRE).

### Fluorescence spectroscopy

Fluorescence measurements were carried out on a Hitachi F-7000 Fluorescence Spectrophotometer. Emission spectra of α-Clu or β-Clu (0.2 mg/ml in PBS, pH 7.4) were recorded with the excitation wavelength set at 295 nm. The hydrophobic probe, 4,4′-Dianilino-1,1′-Binaphthyl-5,5′-Disulfonic Acid (bis-ANS) (Molecular Probes, USA), was used at a final concentration of 10 μM for bis-ANS-binding studies. The samples were excited at 390 nm. All emission spectra were recorded with the excitation and emission band passes set at 2.5 nm. Buffer spectra were recorded and subtracted from protein spectra to obtain the final measurements. All spectra were recorded at a temperature of 25 °C.

### Dynamic Light scattering (DLS)

Hydrodynamic radius (R_h_) of α-Clu and β-Clu (1 mg/ml in PBS, pH 7.4) was determined using Photocor complex DLS system (Photocor Instruments, MD). Protein samples were filtered through a 0.22 μm membrane before making the measurements at 25 °C. A laser of wavelength 632.8 nm was used for collecting the data. The data was processed using DynaLS software (V.2.8.3)

### Sedimentation velocity measurements

Sedimentation velocity measurements were performed using an Optima XL-I analytical ultracentrifuge (Beckman Coulter, Fullerton, CA, USA). α-Clu and β-Clu protein samples at 0.5 mg/ml in 20 mM phosphate buffer (pH 7.4) were subjected to centrifugation at 18,000 rpm at 20 °C, using an An50Ti rotor. The sedimentation coefficient S20,w and molecular mass of the protein were calculated using the program SEDFIT^60^, which uses non-linear regression fitting of the sedimenting boundary profile with Lamm equation,


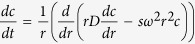


which describes the concentration distribution c(r,t) of a species with sedimentation coefficient, s, and diffusion coefficient, D, in a sector-shaped volume and in the centrifugal field ω^2^r.

### Chaperone activity against amorphous aggregation

The chaperone-like activity of α-Clu and β-Clu was investigated against the heat-induced aggregation of alcohol dehydrogenase (ADH) as well as the DTT-induced aggregation of insulin. The thermal aggregation of yeast ADH (0.2 mg/ml) in the absence or the presence of different concentrations of α-Clu or β-Clu was monitored at 48 °C in 50 mM phosphate buffer, pH 7.2, containing 100 mM NaCl. Aggregation was monitored by measuring light scattering at right angles in a Hitachi F7000 Fluorescence Spectrophotometer. The excitation and emission wavelengths were set at 465 nm and excitation and emission band passes were set at 2.5 nm. DTT-induced aggregation of insulin was monitored in 10 mM phosphate buffer, pH 7.2, containing 100 mM NaCl at 37 °C in the absence or in the presence of α-Clu or β-Clu. The buffer containing required concentrations of α-Clu or β-Clu and a final concentration of 0.2 mg/ml of insulin was incubated for 2 min with constant stirring in a cuvette at 37 °C. Aggregation of insulin was initiated by the addition of 20 mM DTT. Aggregation was monitored by measuring light scattering as described above.

### Cell culture

The cell lines C2C12, F111, A549, COS1, DU145, MCF7, and HepG2, were maintained in DMEM containing 10% Foetal Bovine Serum. 3T3-L1 cells were maintained in DMEM containing 10% Adult Bovine Serum. The cells were expanded to 80% confluency before treatment with α-Clu or/and β-Clu (10 μM) unless otherwise mentioned. For lipid accumulation studies, the cells were grown for 6 days from the start of the experiment and the medium containing the required amount of α-Clu or β-Clu was changed every 48 hours. Cells were trypsinized at the end of the designated time points, washed with PBS and stored at 4 °C until further analysis. For microscopy, the cells were fixed with 10% neutral buffered formalin (NBF) for 10 minutes and washed thoroughly in PBS before staining and visualization.

### FITC Labelling

α-Clu or β-Clu (1 mg/ml in PBS, pH 7.4) were labelled using freshly prepared FITC (0.5 mg/ml) (Sigma, USA) solution in DMSO according to manufacturer’s protocol. Unbound dye was removed using a PD10 column (GE Healthcare, USA).

### Sub cellular localization

Cells at 80% confluency on cover slips, allowed to attach overnight, were treated with media containing FITC-labelled α-Clu or β-Clu. After 24 hours, Lysotracker Red, MitoTracker Red, or ER-Tracker (Life Technologies) was added to the cells at a final concentration of 1 μM and incubated for 10 minutes followed by extensive washing, fixation and visualization with the Leica TCS SP8 confocal platform. Cover slips were mounted using Vectashield mounting media (Vector Laboratories Inc, USA) containing DAPI for visualization of nuclei.

### Neutral lipid visualization

Neutral lipids accumulated in cells were stained with either Oil Red O (ORO) or Nile Red. Fixed cells were washed with PBS and incubated with freshly prepared ORO-isopropanol (60%) for 1 hour at room temperature. Subsequently, cells were washed with 60% Isopropanol and PBS and visualized on a Carl Zeiss Axiovert 200 M inverted fluorescence microscope. Alternately, Nile Red (100 μg/ml in acetone) was added to the cells at a 1:100 dilution in PBS and incubated for 10 minutes. Cells were washed thoroughly and visualized under the confocal microscope.

### Quantification of lipid

Cells were incubated with Nile red (100 ng/ml in PBS) for 10 minutes, washed twice and re-suspended in PBS for flow cytometric analysis. BD FACS Calibur was used for measuring cell-associated Nile red fluorescence (excitation - 488 nm, 570/20 nm band pass filter). Cells were gated on forward scatter to exclude cell debris and 10,000 gated events were recorded. Data was corrected for cell-associated auto fluorescence and analyzed using the Cell Quest software.

### Animal studies

All experimental protocols were approved by the Institutional Animal Ethics Committee (IAEC) of the Centre for Cellular and Molecular Biology, Hyderabad (IAEC clearance number for the project- 62/2013), in accordance with the guidelines of the CPCSEA (Committee for the Purpose of Control and Supervision of Experiments on Animals), Ministry of Environment, Forest and Climate Change, Government of India. Animal studies were performed in accordance with the Institutional Animal Ethics Committee norms. Four month old, male, New Zealand white rabbits were taken in 3 groups: control (n = 8), β-Clu-injected (n = 8) and α-Clu injected (n = 8) animals. α-Clu or β-Clu (500 μg), emulsified with adjuvant, was injected subcutaneously thrice at intervals of 15–20 days. Animals were weighed regularly. At the end of 12 months, all surviving animals were sacrificed. Organs isolated from them were fixed in 10% NBF, dehydrated and embedded in paraffin wax. Haematoxylin and eosin staining was performed on 4 μm tissue sections visualized using Axioplan 2 Inverted Fluorescence Microscope (Carl Zeiss Microscopy, USA). Images were acquired using Axiovision software.

## Additional Information

**How to cite this article**: Matukumalli, S. R. *et al*. Clusterin: full-length protein and one of its chains show opposing effects on cellular lipid accumulation. *Sci. Rep.* **7**, 41235; doi: 10.1038/srep41235 (2017).

**Publisher's note:** Springer Nature remains neutral with regard to jurisdictional claims in published maps and institutional affiliations.
